# Let’s Campaign for a Fairer Society in the Aftermath of COVID-19

**DOI:** 10.3389/fsoc.2021.789906

**Published:** 2022-02-04

**Authors:** Graham Scambler

**Affiliations:** Department of Sociology, University of Surrey, Guildford, United Kingdom

**Keywords:** austerity, rentier capitalism, COVID-19, fairness, sociology, good society

## Abstract

In this paper I ground a brief account of the impact of COVID-19 on the United Kingdom in an understanding of a decade of austerity politics from 2010 to 2020, itself a product of the advent and consolidation of post-1970s financialised or rentier capitalism. I argue that such an analysis is essential if realistic plans are to be laid for a “better”—understood here as a more equitable or “fairer”—society. I go on to consider the contributions that sociology can, and arguably should, make to this end. This involves a range of engagements from scholarship at one end of the spectrum to action or muckraking sociology at the other. In addition to plotting a role for sociology, I suggest a set of criteria for recognizing a “fairer society”; postulate a series of institutional reforms that might characterize the attainment of such a society; and outline and confront social structural, cultural and agential obstacles to its realization. A theme running throughout the paper is that the delineation and promulgation of the “good society” remains central to any credible—that is, post-Enlightenment reconstruction of - the sociological project.

## Introduction

COVID-19 (hereafter COVID) has hit the United Kingdom (UK) harder than it has many other kindred western, post-industrial societies. There is an emerging picture on why this is which can be encapsulated in the following propositions:

• the UK was ill-prepared for a pandemic, having paid too little attention to prior warnings about shortages of hospital beds, equipment and facilities;
• the context of this pandemic-specific lack of readiness was a health system, the National Health Service (NHS), that had been systematically under-funded during a decade of political austerity from 2010–2020;
• austerity was a calculated political choice that reflected a governmental agenda to open up public sector institutions, including the NHS, to competition from private business;
• once COVID entered the UK, and even allowing for the lack of readiness, the governmental response was inadequate in terms of: delayed action, strategic errors, inefficiency and loss of trust;
• COVID has provided prolonged cover both for the assault on the NHS and for other political initiatives;
• politically, COVID has permitted the systematic pursuit of state authoritarianism.

I am aware that this series of assertions will be regarded by some as: 1) polemical rather than sociological; 2) in need of more by way of empirical corroboration; and/or 3) signaling a form of activism or engagement in the volatile contemporary politics of the UK. I will not defend them again as I have done so in some detail elsewhere (Scambler, 2020; Scambler et al., 2021; and in numerous blogs at: www.grahamscambler.com; see also Ashton, 2020; Horton, 2020; Sample, 2021). My focus in this paper is “society after COVID”. More particularly, I want to address how COVID has revealed the true nature of our “fractured society” in the UK (Scambler, 2018b); the primary structural, cultural and agential obstacles to the creation of a better—interpreted here as a “fairer”—society; and the role that sociology might play in this process. It might be objected that an analysis based on the nation state in general, and a single example like the UK in particular, is no longer compelling in a globalised world; but the intention in this paper is to start the ball rolling and in the process to exhort further theory and research based on a comparative perspective.

## The Fractured Society

There are multiple ways in which UK society might be judged fractured, and in which COVID can be said to have exposed, widened and deepened these fractures. Moreover it is crucial that any comments on post-COVID society are rooted in an understanding of the “take-off” point that is the present. Rather than repeat more comprehensive comments from prior publications on the nature and extent of extant fractures, I will here settle on a few select themes. While the most threatening of the fractures, the ineluctable culmination of what (Beck, 1992) termed the “boomerang effect” in his *Risk Society*, is undoubtedly climate change, and the most immediate the global pandemic, the thematic focus of this paper will be on intra-national inequality, cultural relativity and the generative mechanisms of what I have called the “class/command”, “stigma/deviance”, “insider/outsider” and party/populist’ dynamics (Scambler, 2020a).

## Inter- and Intra-National Inequality

Increasing intra-national inequality in the UK is associated with the transition from industrial to post-industrial or rentier capitalism in the 1970s. The differences in employment and taxation systems in industrial and post-industrial capitalism are summarized in Box 1. Rent is defined in this context as income derived from the ownership, possession or control of scarce assets under conditions of limited or no competition (Christophers, 2020; see also; Sayer, 2016). Significant—and often high - levels of wealth and income inequality have been a feature of societal formations throughout history. If the period of Occidental welfare statist capitalism following the conclusion of WW2 was exceptional, providing something of a “lull”, inequality has once again grown substantially in rentier capitalism post-1980s. Moreover the arrival of COVID has witnessed a conspicuous spurt. Jack Leslie, speaking for the Resolution Foundation, is quoted as saying that it is unusual for wealth to increase during a recession but that the impact of events during 2020 and 2021 had “turbo-charged” the gap between rich and poor (see Elliot, 2021).

Box 1 **The Principal Differences in Employment Relations and Taxation Systems in Industrial and Post-industrial Capitalism.**

Source: Byrne and Ruane (2017).

**Table:** 

Industrial employment relations and tax system	Post-industrial employment relations and taxation system
Keynesian/Beveridge mode of regulation	Post-industrial/consolidation state mode of regulation
Industrial workforce approaching half of total workforce	Primarily service sector workforce; industrial workforce less than 15% of total
Full employment with frictional unemployment	Disguised unemployment (e.g., extension of higher education; early retirement); underemployment
Job security and substantial worker rights	Flexible labour—spread of precarious employment, limited worker rights
Employer-borne risk and responsibilities to workforce	Transfer of risk to workers—use of 0 hours contracts and forms of self-employment
Large public sector and devalorised labour	Declining public sector as proportion of all employment and recommodification of labour
Relatively high trade union membership	Low trade union membership
Relatively high wages	Lagging wages and spread of low wages; heavy reliance on wage subsidy
Strong protections for workers in public sector	Workers in public sector exposed to market competition
Status and protection for professionals	Extension of Fordism into professional work and proletarianisation
High top rates of income tax	Relatively low top rates of income tax
Relatively strong link between national insurance contributions and benefits received	Weak link between national insurance contributions and benefits received
Higher corporation tax rates	Lower corporation tax rates
Avoidance and evasion practices which do not catastrophically compromise the tax system	Avoidance and evasion practices which catastrophically compromise the tax system
Strong and independent tax collection authorities	Weakened tax collection authorities strongly influenced by corporate lobbying

The UK experienced its biggest 1-year fall in output in over three centuries in 2020, but since the start of the crisis in February 2020 total household saving rose by £200bn, household debts (excluding credit cards) decreased by approximately £10bn, and house prices - which fell by an average of 22% over the previous four recessions—rose by 8%. Overall, total UK wealth increased by £900bn to £16.5tn during the pandemic, but the poorest households were more likely to have run down rather than increased their savings. Nor did they share in the house price boom because they were less likely to own a home. The richest 20% of households were four times as likely to have increased their savings during the crisis as the poorest 20% of households (47 vs. 12%), and 2.5 times as likely to have reduced their debts. According to the Resolution Foundation, this reflected pandemic-induced spending reductions being concentrated among those on higher incomes. The adults in the richest 10% of households currently have wealth of £1.4m each following a £50,000 increase during the crisis, while the poorest 30% gained an average of just £86 per adult in additional wealth.

A further brief comment on wealth distribution is relevant, and it concerns that fraction of the top 1% of wealth-owners, the “super-rich”, whom I have long argued play a crucial role in producing and reproducing inequality in the UK (Scambler, 2018b). According to the latest annual *Sunday Times* listing, an additional 24 billionaires were added to the UK’s list during 2020/21, making a total of 171, the highest number in the 33 years of the newspaper’s rich lists. The gains reaped by the super-rich came in a year that the UK government stepped in to pay the wages of millions of citizens (see Jolly, 2021).

What of income inequality? Unsurprising, in fact with unerring predictability, income inequality in the UK too grew both during the decade of political austerity and with the arrival of COVID. According to the Office of National Statistics (Office of National Statistics, 2021), income inequality had steadily increased to 36.3%—as measured by the Gini coefficient—just before the pandemic. This was the highest level of income inequality over the previous decade (if lower than levels reported during the economic downturn of 2008). While income inequality for people in retired households increased 3.5 percentage points to 30.7% in the decade ending in 2020, income inequality among those in non-retired households increased on average by 0.2% per annum to 36.5% over this same period. Interestingly, the typical working-age income level in the UK is £29,437 while in France it is £29,350 (Savage, 2021); however, the poorest fifth of working-age households in the UK are 20% poorer than their French counterparts, while the richest fifth are 17% richer. Overall, the combination of lower incomes at the bottom level of British earners, comparatively low levels of private savings, and a less generous security safety net has meant that UK households were particularly exposed to economic shocks like the COVID crisis.

The incomes of top earners followed a different pattern. The annual pay of FTSE chief executives actually fell during the pandemic but still equates to what a “key worker” would earn in a lifetime (High Pay Centre, 2021). The CEOs of companies in the blue-chip share index were paid £2.69m on average in 2020, with vaccine-maker AstraZeneca’s chief executive taking the top place courtesy of a £15.45m deal. The average CEO pay figure fell by 17% compared with the £3.25m recorded in 2019 but is still 86 times what an average British worker earns in a year. Interestingly, across the nine companies that used the furlough scheme for their employees but have yet to repay it, CEOs earned an average of £2.2m each.

So wealth and income inequality, exacerbated by COVID, remain distinctive and significant in the UK. Health inequalities have a similar, and causally related, profile. While changes to employment relations and the tax system outlined in Box 1 predictably enhanced health inequalities, research published in 2017 estimated that spending cuts in health and social care during the decade of austerity also contributed to an excess of 45,000 deaths between 2012 and 2014, and would lead to approximately 150,000 further excess deaths between 2015 and 2020 (Watkins et al., 2017). Marmot et al. (2020), updating their review of ten yours earlier, found that people can now expect to live more of their lives in poor health; improvements in life expectancy have stalled, and declined for the poorest 10% of women; the health gap has grown between wealthy and deprived areas; and living in a deprived area in the North East is worse for people’s health than living in a similarly deprived area in London, to the extent that life expectancy is nearly 5 years less (see also Hiam et al., 2020). The advent of COVID, predictably and unquestionably, has further exacerbated health inequalities along predictable lines, notably around class, ethnicity or race and disability (Marmot and Allen, 2020; Bambra et al., 2021).

## Cultural Relativity

A causal companion of rentier capitalism—although by no means either determined by or reducible to it—has been a discernible cultural shift. This has been characterised in numerous different if overlapping ways. The umbrella term of “cultural relativity” is designed to capture the shift in all its variety. As Lyotard (1984) maintained a generation ago, “grand” narratives (embracing ideas of progress, whether pro- or anti-capitalist) have given way to a multiplicity of “petit” narratives (offering people a bewildering array of “choices” of beliefs, attitudes, philosophies and lifestyles). This latter has been analysed using concepts like recognition and belonging (see Honneth, 2020); and it has given rise to a debate between theorists who continue to emphasise social structure in general, and social class in particular, and consider the contradictory and competing material interests of capitalists and wage labourers to be the fundamental division in rentier capitalism; and theorists who argue that the cultural shift has displaced, compromised, skewed, or at least diminished, structural class relations. There is clear evidence that the contemporary shift in culture has impacted significantly on people’s “subjective” sense of their own class position and interests. Houtman et al. (2012), for example, show how throughout Europe people’s voting behaviour no longer “simply” follows class location. They rightly conclude, however, that “class is not dead, but has been buried alive under the increasing weight of cultural politics” (Houtman et al., 2012: 121). Class as an “objective” structural factor, I too have contended, remains alive and well, indeed the more so in rentier capitalism than in welfare state capitalism, hence the sustained growth in wealth, income and health inequalities (Scambler, 2018b).

More needs to be said on cultural relativity than that it occludes and obscures the *heightened* causal efficacy of objective structural relations of class for personal health, wellbeing and longevity. Just as (Habermas, 1989) was surely correct to insist that a postmodern culture—because it denies the possibility of a rationally compelling alternative to the status quo—constitutes a “new conservatism”, so the cultural shift alluded to here is a very real threat to any potential for transformative social change. This is so for reasons beyond a resistance to constructing “grand” narratives for change. An analysis of cultural relativity beyond Lyotard’s postulation of “grand” vs. “petit” narratives suggests that something akin to a “grand” narrative has now emerged *at the end of “grand” narratives*. Allied to the contemporary strands of populism, this can be defined in terms of a family of concepts that include “post-truth”, “anti-woke”, “culture clash”, “cancel culture”, “gaslighting”, “hate speech”, and so on. They find their personification in politicians like Trump and Johnson. The point to note is that politicians for whom the status quo remains desirable have re-jigged today’s “petit” narratives to construct what, after (Lakatos, 1970), might be called a “protective belt” of auxiliary “cultural theories” around the core of their neoliberal ideology.

## Class/Command, Stigma/Deviance, Insider/Outsider and Party/Populist

It is one thing to document and theorise structural and cultural change, and quite another to account for or to explain it sociologically. Four generative mechanisms (among many) are proffered here (see also Scambler, 2020a; Scambler, 2020b). The first is the “class/command dynamic”. This asserts that a fraction of the 1% called out by the Occupy Movement, those I have called “capital monopolists” (ie financiers, major shareholders, CEOs of transnational companies), the essence of the contemporary transnational and nomadic “ruling class” in more familiar parlance, now exercise greater sway over the national state apparatus than was the case in welfare state capitalism: capital buys power to make policy in its interests and now gets more for its political investments [as has become particularly apparent during COVID (see Scambler et al., 2021)]. The pandemic has proved hugely profitable for capitalists in general and capital monopolists in particular (Preston and Firth, 2020). I have made what I think is now an uncontroversial case that the class/command dynamic is the prepotent generative mechanism for understanding and explaining the emergence and impact of rentier capitalism.

The “stigma/deviance dynamic” is one of a number of companion mechanisms (Scambler, 2020a). It maintains that shame (stigma) is being increasingly and strategically converted into blame (deviance) for political ends. Employing (Goffman, 1968) terminology, people once seen as “non-conforming” are being recast as “non-compliant”. Just as it takes power to effectively police shame, so it does to append blame to shame (Scambler, 2018a; Tyler, 2020). “Heaping blame on shame”, or “weaponising stigma”, has the effect of rendering those subjected to this strategy “abject”; and people who are abject find it very difficult to resist policies and practices instituted to their disadvantage. The enhanced rate of class exploitation that has simultaneously lit and stoked the fires of capital accumulation and deepened wealth, income and health inequalities has been facilitated by the state, and the stigma/deviance dynamic has been a significant device to this end. If people can be blamed for their shameful difference, then they can more easily be abandoned by the state, leading to the cutting of tax-funded welfare expenditures.

The third dynamic is that of the “insider/outsider”. Rentier capitalism’s cultural relativism is pertinent here. It has led to cultural disorientation, a “disconnected fatalism” and a tendency to fundamentalist thinking and populist engagement. The diminution of the role of structures like class as primary resources for identity formation, together with the emergence of identity politics, has prompted new levels of cultural disinhibition. Othering, or “outsidering”, has become easier. Adapting (Elias and Scroton, 2008) distinction between the established and outsiders, Pentintseva (2015) delineates four dimensions to processes of outsidering with special reference the contemporary movement of peoples:

• the relatively powerless position: in economic terms, but also in terms of access to social or formal facilities, services or institutions (e.g., legal status, possibilities of mobility, status differentials in institutional contexts);
• the lack of protection and opportunities afforded by membership in powerful social networks;
• limited internal cohesion between new migrants as a whole, rooted in the fact that they are all “new”;
• representations and stereotypes of these groups as threatening, images based on the socially unacceptable characteristics of a small minority of group members (eg issues of social distancing, “ethnicising” and problematising particular attributes).

There is ample evidence in the UK and throughout much of the European Union that the insider/outsider dynamic, allied with the stigma/deviance dynamic, both involving a politics of othering, have led to a widespread recasting of refugees and asylum seekers as parasitic economic migrants. It is a phenomenon with an unambiguous genesis in imperialism, colonialism and racism (Tyler, 2020). Thus the insider/outsider dynamic has a strong ethnic or racial bias (e.g., May’s “hostile environment” policy and the “Windrush” scandal).

The “party/populist” dynamic has to do with the class de-alignment in party political voting in the UK, which is in part a by-product of the “intrusion” of cultural issues. But in the UK as elsewhere formerly stable and secure mainstream political parties are being challenged by putative populist movements. Fraser (2019) argues that a pre-existing “hegemonic bloc”, articulated in the form of “progressive neoliberalism”, has recently given way to a period she classifies as an interregnum. She distinguishes between “reactionary populism” and “progressive populism” as current contenders for dominance. Given that the UK’s Conservative Party under Johnson—that ex-Eton and Oxford “boy/man” (Beard, 2021) - has moved to subsume, and to a degree represent, the racialised reactionary populism much in evidence around Brexit, plus the ongoing divisions in the Labour Party post-Corbyn, an element of neoliberal governmental stability and security, however precarious, has seemingly returned.

There is no contention here that this quartet of dynamics exhausts the social structures that lie behind contemporary social order/change, merely that they are playing major roles. What should be clear is that any attempts to “build back better”—to reiterate, *understood as fairer—*will have to overcome an intimidating set of structural, cultural and agential obstacles.

## The Sociological Project

I see the sociological project as part and parcel of Habermas’ “reconstructed” Enlightenment project, that is, of a re-grounded Enlightenment project severed from its white, imperial, male and classed roots (Habermas, 1989; Scambler, 1996). It issues in the close-knit family of six types of sociology, sociologist and logics or modes of engagement outlined in Box 2. The first four will be familiar from the seminal paper by Burawoy (2005). Of my additions, *foresight sociology,* associated with what I have called a speculative mode of engagement, refers to the anticipation of alternate futures, while *action sociology*, strategic in orientation, refers to what I argue is a collective sociological responsibility to counter *and overcome* ideological misrepresentations of the social world emergent from vested interests. In what follows the emphasis is on these two, as yet under-represented, types of sociology (see Scambler, 2018b). The account on offer claims to be: 1) consonant with the latest empirical research conducted within professional sociology; 2) a challenge to the lack of ambition often found within policy sociology; 3) anchored in a critical sociology of contemporary societal change; and 4) an extension and development out of public sociology.

Box 2 **| Six Types of Sociology.**

**Table:** 

Sociologies	Sociologists	Mode of Engagement
Professional	Scholar	Cumulative
Policy	Reformer	Utilitarian
Critical	Radical	Meta-theoretical
Public	Democrat	Communicative
Foresight	Visionary	Speculative
Action	Activist	Strategic

Source: Scambler and Scambler (2015).

Greener (In Press) affords a useful starting point for the application of the sociological project when he revisits the Beveridge Report of 1942 (Beveridge, 1942) and its examination of the societal ills of the time and its proposed remedies. Beveridge, it will be recalled, discerned five “Giants’”that required tackling: Want, Disease, Ignorance, Idleness and Squalor. Greener argues that this quintet might now be replaced by five “New Giants”:

• Inequality: this could be seen as a replacement for Want. What is different between 1942 and the present is that despite the fact that on average people are considerably richer, there are growing differences in wealth and income between social strata. “Inequality is a New Giant because individually we can do little about it—it requires a collective solution. Rising inequality appears to be a symptom of an increasingly dysfunctional form of capitalism … Rising inequality, in a time of lower economic growth than that which was achieved in the 1950s and 1960s means all boats do not rise together, and flat living standards for some while others become wealthy at levels not seen since the 1930s” (Greener, In Press: 8).
• Preventable mortality: this can be seen as a replacement for Disease. COVID has reaffirmed that inequalities have profound links to health. Moreover, preventable mortality is another “collective action problem”, requiring both structural inequalities and the nature and reach of the health system to be addressed.
• Job quality: Beveridge maintained that people out of work, or Idle, were particularly likely to fall into poverty. The present job market is very different from that in 1942: the Fordism of industrial society has been replaced by the less unionized and well-remunerated service sector jobs of post-industrial society (see Box 1). Households are much more likely to have all adults working … Work has become more precarious since the 1980s leading analysts of change writing of the “precariat …” (Greener, In Press: 9). Furthermore the future looks daunting as robots and artificial intelligence algorithms wait in the wings.
• Fragmenting democracy: Greener selects this from a number of possible replacements for Ignorance. For all the recent focus on education levels, he detects “a sense of democracy being in crisis” which he connects with the rise of populism. In particular: “online privacy is being compromised and used to tailor political messaging, as well as to spread untruth … ”(Greener, In Press: 10). Ignorance has taken on a new look in association in the era of cultural relativity.
• Environmental degradation: whereas Beveridge was concerned above all with housing when he considered Squalor, and notwithstanding continued housing travails, “there is a much bigger threat attached to the circumstances in which we live—that of environmental degradations and the problems it will bring to us all” (Greener, In Press: 11). COVID assumes a somewhat lesser salience in the context of climate change.

Greener’s analysis naturally invites an interrogation of the role of sociology in responding to New Giants such as these. Three challenges stand out: 1) what might a “good”—or better, fairer—society look like; 2) what are the major obstacles to its creation; and 3) how might these obstacles be overcome. Each will be commented on in turn.

### Parameters for a Fairer Society

Beveridge was of course not a sociologist, but rather an evidence-based reformer, committed to improving the lot of the citizenry as a whole in the wake of WW2. He provided a policy blueprint and something of a narrative without drifting into utopianism. The notion of foresight sociology introduced earlier exhorts a sociological engagement with parameters for alternative futures, extending to detailed innovative institutional change. As Levitas (2017): 3) maintains, “the imagination of a potential, different society in the future draws attention to the need for change, offers a direction towards that change, and a stimulus to action in the present.” (see also Levitas, 2013). This is not the occasion to go into detail, but a few preliminary comments are in order.

The first point is to acknowledge that those philosophical principles of democracy, freedom, equality, justice and so on that commonly constitute criteria for identifying a good society are independent but not absolute: there is necessarily a trade off between them in any actually existing societal formation. The telling query thus becomes: are the trade offs optimal for the citizenry as a whole? In the UK, as in many other societies, there are conspicuous and troubling deficits. Consider democracy for example. The evidence is unambiguous. Miliband (1965) argued long ago that the UK’s parliamentary democracy might more accurately be called a “capitalist democracy” in that it functions to underpin and reproduce the vested interests of a tiny minority. Explicated in terms of the four generative mechanisms outlined earlier, a strong case can be made that the class/command dynamic subverts democratic policy-making by privileging and facilitating the capital accumulation of a fraction of the 1%; the stigma/deviance dynamic assists in this process by silencing or marginalising the voices of those most vulnerable to (class-based) exploitation and/or (command or state-based) oppression; the insider/outside dynamic further “others” the marginalized whilst adding a strong racialised element - most dramatically captured in the relabeling of refugees and asylum seekers as “economic migrants”—which diverts public attention from exploitative and oppressive measures; and, finally, the party/populist dynamic allows for the translation of “othering” into an electoral asset. When added to this structural mix, the phenomenon identified here as cultural relativity makes the construction and dissemination of narratives for social change and resistance to the political status quo all the more difficult.

It is implicit in the concept of trade offs that subversions of democratic processes have echoes, and themselves echo, compromises in other principles. A democratic deficit, for example, will almost invariably be linked with deficits in freedom, equality and justice. To resurrect an old liberal distinction between formal and actual freedom, it is apparent that while all citizens are formally free to apply to send their offspring to Eton, still the breeding ground for multiple UK elites, very few are actually free to do so: they simply lack the requisite economic, cultural and social capital. The extent of wealth and income inequality in UK’s rentier capitalism was sketched earlier. Nor can there be any doubt that this family of deficits in democracy, freedom and equality add up to a substantial charge of injustice. It is not just that in the UK the trade offs between independent political principles has gone seriously awry, but also that no lasting remedy can be accomplished via pro-capitalist piecemeal social engineering, which is all that capitalist/parliamentary democracy can offer [and that only when confronted by a potential crisis of legimation (Habermas, 1975)]. It will require a social transformation to re-set the UK’s family of interlinked principles of good governance.

### Obstacles to Creating a Fairer Society

For all that a number of professional sociologists are on record as anticipating a likely crisis in rentier capitalism (see Wallerstein et al., 2013; Streeck, 2016), it is axiomatic both that capitalism possesses remarkable powers of recovery and reinvention and that the structural, cultural and agential obstacles to its displacement are considerable. The significance of structural obstacles in this regard was implicit in the earlier discussion of the class/command dynamic for example, and further elaboration is unnecessary at this juncture. People in the UK inhabit a social world structured by sex/gender and race/ethnicity as well as by class. Indeed, for all that it is objective class relations that remain pivotal for capitalism, capitalism has from its origins in the long 16th century wrested advantage from pre-existing structures around sex/gender and race/ethnicity. Moreover, as intersectional studies have demonstrated, the boundaries in lived experience between the working class, women and racial minorities have long been and remain porous.

The cultural shift towards relativity has readily accommodated individualized discourses around identity politics and human rights. These are of course important matters in their own right. However, Sayer (2005): 87) makes the important point that capitalism is not dependent on identity; and he goes on to assert that one of the great disappointments of recent research on inequalities has been “a tendency to invert the former neglect of identity-sensitive, cultural influences by denying the co-presence of identity-neutral mechanisms.” To rehearse a view outlined earlier, subjective class relations may have diminished in salience for identity formation, but objective class relations (articulated *via* identity-neutral mechanisms) have certainly not (see Scambler, In Press).

These cultural phenomena have significant implications for mounting challenges to the status quo. While rentier capitalism could conceivably implode as a result of its own largely unopposed excesses (see Wallerstein et al., 2013), cultural relativity: 1) inhibits the construction of rival “grand” narratives proclaiming alternate futures, and thus 2) undermines both a collective sense of solidarity and the potential for the collective “agential” action that Greener and others argue is a prerequisite for effective social change.

### Overcoming Obstacles to Transformative Change

Even allowing for the possibility of a “self-destruct”, the historical durability of capitalism and the severity of the structural, cultural and agential obstacles to achieving transformative change are daunting. Wright (2019) discerns a sextet of “logics”:

• Smashing capitalism: the classic logic of revolutionaries, maintaining that capitalism is unreformable and must be destroyed and replaced by socialism;
• Dismantling capitalism: the logic of that subset of revolutionaries who eschew the notion of “rupture”—with all its “unpredictable” sequelae—in favour of state-directed reforms that incrementally introduce a socialist alternative from above;
• Taming capitalism: this logic commends neutralizing the “harms of capitalism” without replacing it;
• Resisting capitalism: the logic of opposing capitalism from outside of the state and without the motivation and ambition of capturing state power;
• Escaping capitalism: the logic here is that capitalism is too powerfully entrenched to overthrow but that insulation from its harms is possible, for example in sheltered or cooperative communities.

Wright’s exhorts an amalgam of logics, which he entitles “eroding capitalism”. No economy, he contends, is “purely” capitalist, and it is always possible to build more democratic, egalitarian, participatory economic relations in the cracks and fissures of the fractured society. Such initiatives might in the long term lead to the displacement of rentier capitalism’s systematic, economic role in society.

The strategy of “permanent reform” espoused elsewhere might perhaps be cast as “escaping capitalism” plus “resisting capitalism” through “dismantling capitalism” towards “smashing capitalism” (see Scambler, 2018b). The thinking behind it can be summarized as follows:

• Social structures like class, gender and race, understood as enduring (beneath-the-surface) mechanisms, have long historical tap roots and are exceptionally resistant to (on-the-surface) agential efforts to revise, deconstruct or disassemble them.
• Within the UK’s existing system of capitalist democracy, a parliamentary route to transformatory structural change is all but inconceivable, as was evidenced recently by the rapid and effective undermining of the Corbyn challenge.
• Principal among the mechanisms causally responsible for wealth, income and health inequalities is a class/command dynamic, reinvigorated in the period of rentier capitalism, which asserts that these inequalities derive above all else from the transnational sway of a tiny minority of owners of capital assets who buy power from the nation state to implement policies to their advantage.
• Given the ongoing “contamination” of the state’s power elite and the impotence of parliament, the only way to effect structural, transformatory change is by mobilizing the populace (Della Porta, 2020).
• A precondition of the effectiveness of a people’s movement is that it is class-based, in other words underwritten by the working class, which as Wright maintained, unites the bulk of those who “work to live”.
• Although the likelihood of this kind of working-class unity and class-based collective action—and hence of an effective people’s movement—remains slight, it can (perhaps only) occur in the event of a “trigger event” occasioning a crisis of state legitimation.
• Such a people’s movement will necessarily involve a series of alliances across overlapping campaigns and group interests.
• As Bambra and colleagues (2021) have suggested, COVID might carry the potential to precipitate a legitimation crisis (see also Tooze, 2021): transformatory change typically only obtains after a major social upheaval, as occurred in the UK after WW2.
• The optimal strategy in present circumstances could therefore be one of permanent reform, that is, a continuous and coordinated push for reform along a spectrum from the *attainable* to the *aspirational* (e.g., from local municipal and/or cooperative initiatives to those that expose and call into question enduring social structures).

### Permanent Reform

It is one thing to prescribe a strategy and another to show what it might look like in practice. In this section, after a few words on culture, the focus is on attainable-to-aspirational economic change. Privilege and advantage are written into and underlined in UK culture. This is nowhere more apparent than in the survival of a monarchy that remains obscenely rich and clandestinely and extra-legally active in the political arena. Why is it important? Because its absence would expose to critique a series of institutions that are parasitic upon it, like hereditary titles, the House of Lords and the honours system. When the present monarch dies, attainable ends might include a trimming of “the firm”, the abolition of hereditary titles, the replacement of the House of Lords by a second chamber based on deliberative democracy and the end of the conspicuously corrupt honours system [a quarter of top Conservative Party donors have received honours or peerages]. Aspirational change might include the introduction of republican governance. The point to emphasise here is that culturally and economically attainable reforms en route to aspirational or transformatory change are related dialectically and, arguably, need to occur in tandem.

Of overriding import are challenges to rentier capitalism itself. Towards the attainable end of the spectrum might be included such measures as restoring trade union rights, abolishing hire and rehire practices, ending zero hour contracts, increasing sick pay, replacing Universal Credit, raising the UK’s parsimonious state pension, restoring funding to the NHS, and expanding public housing. Christophers (2020) is particularly helpful as we move towards the aspirational end of the spectrum (see here Sayer and McCartney (2021) on the importance of attainable-to-aspirational reforms for reducing health inequalities). Following his detailed examination of the UK’s overriding commitment to rentier capitalism, he considers several options for policy shifts. The first of these involves competition policy, and his focus is on countering the negative consequences of pervasive monopoly so often facilitated by the state: “unconstrained” capitalism tends towards the monopoly conditions favoured by its most powerful actors’ (Christophers, 2020: 388). Towards the attainable end of the permanent reform spectrum might be changes to a tax system that currently betrays an extreme “rentier bent”. Reforms to modify the system so as to limit rentiers’ ability to make excess profits might, for example, reduce the extant tax-based subventions supporting rentiers as well as increasing tax rates. Tax havens should clearly be closed.

Piketty (2014) has estimated that the return on assets (*r*) globally before tax has always been greater than the rate of economic growth (*g*); and for most of the history of capitalism, *r* after tax has also been greater than *g*, hence his claim that, *ceteris paribus*, wealth inequality increases under capitalism. Unusually during the period after WW2, *g* exceeded net (post-tax) *r*, in the process curbing inequality (largely through a combination of “exceptional growth” and progressive taxation policies). In the present era of rentier capitalism Piketty commends higher taxes on assets to bring *r* back below *g* and thus counteract surging wealth inequality (Christophers, 2020: 392). In similar vein, Monbiot (2018) argues for breaking the power of “patrimonial capital” and the vicious circle of wealth accumulation and inequality.

Christophers adds that UK governments have not only “featherbedded rentiers”, they have actively encouraged them, *via* tax subsidies, to be/become rentiers. Taxes on incomes earned from non-rentier activities could be lowered, he advises, “utilizing what economists refer to as the “negative reinforcement” aspect of taxation: removing an aversive stimulus in order to strengthen what is deemed to be a positive behaviour or outcome”; and taxes on rentier assets and income streams could be introduced, or increased, thereby discouraging rentierism (he gives the example here of introducing a land-value tax) (Christophers, 2020: 393).

Christophers also promotes the idea of a state investment bank, which would have the potential to contribute to seeding/funding a transition away from rentierism. Unarguably, this transition requires to be accompanied by another: to a low-carbon future. So what kind of salient non-asset intensive economic activities might the state invest in? Christophers’ shorthand answer is workers and their skills rather than non-human assets and their protection from competition. He appends to this investment in collective consumption of essential or “foundational” goods and services, including material services (e.g., pipes and cables, networks and branches distributing water, electricity, banking services and food), and providential services (e.g., education, health and social care and income maintenance). Another necessary and vital state investment, to reiterate, is in a green or carbon-neutral economy.

Wrapping up this brief sketch of attainable-to-aspirational economic policy change, special reference needs to be made to ownership. For any meaningful transformation from rentier capitalism, the current direction of ownership transfers from the public to the private sector must be reversed. This means shrinking the portfolio of exclusive proprietary assets on which the rentier is able to “earn” private rents. Monbiot (2018): “the economic power of the owners of wealth translates into political power. The richer a tiny segment of society becomes, the better it is able to capture politics and undermine democracy. Eventually, we get a government of the elite, by the elite, for the elite.” This, Christophers accurately concludes, is the current state of play in the UK.

## Conclusion: Sociology and the Fractured Society

This paper has covered a lot of ground, and necessarily in an abbreviated fashion. No apology is offered for this since the objective was to set out, with examples, a framework for a vibrant, relevant and engaged sociology of the UK as a fractured society facing something of a perfect storm of political austerity, Brexit, COVID and climate change in an era of largely unopposed post-industrial rentier capitalism. While there are the beginnings of a professional macro-sociological literature on these issues, there remain deficits in both a foresight sociology of alternate—utopian, dystopian or merely revised—futures and an action sociology of commitment to the pursuit of a good or better, fairer, post-fractured society. As Christophers concludes in his excellent analysis of rentier capitalism, the obstacles—structural, cultural, agential, causal offspring of the quartet of “dynamics” outlined earlier—remain daunting. He notes the strength of the “establishment backlash” against the proposals mooted by the Corbyn-led Labour Party during the course of 2017 and 2019. Witness for example the proposals for progressive land reform contained in the Labour-commissioned “Land for the Many”, published in mid-2019. Monbiot, one of its co-authors, was led to reflect on the readiness of the UK’s “billionaire press” to go into battle on behalf of the ‘ultra-rich’ and “oligarchic power” (Christophers, 2020: 415).

It is critically important that sociologists address these issues: doing so does not turn them into polemicists, although it may involve contesting institutional pressures, which can amount to a form of sociological taming (Scambler, 1996; Scambler, 2018b). While it remains a legitimate and important aspect of the sociological project: 1) for professional sociologists to conduct research into a broad and “unlimited” range of social phenomena, 2) for policy sociologists to explore issues with a view to informing exercises in piecemeal social engineering, 3) for critical sociologists to remain reflective about the evolving paradigms that constitute disciplinary practices, and 4) for public sociologists to disseminate its theories and research into the public sphere of the lifeworld, I have suggested that this cannot/should not satisfy the extent of its responsibilities. This paper has taken the sociology of the COVID pandemic as a starting point and outlined, with illustrations, an agenda that deliberately encompasses classical sociological issues of social order and social change whilst insisting that it should - after Habermas—commit to a “reconstructed” Enlightenment project and extend its remit to advocate for what was once termed the good society.

## Data Availability

The original contributions presented in the study are included in the article/Supplementary Material, further inquiries can be directed to the corresponding author.
